# Analysis of the Relative Humidity Response of Hydrophilic Polymers for Optical Fiber Sensing

**DOI:** 10.3390/polym14030439

**Published:** 2022-01-22

**Authors:** Bernardo Dias, João Carvalho, João P. Mendes, José M. M. M. Almeida, Luís C. C. Coelho

**Affiliations:** 1 INESC TEC—Institute for Systems and Computer Engineering, Technology and Science, and Faculty of Sciences, University of Porto, 4169-007 Porto, Portugal; up201704015@edu.fc.up.pt (J.C.); joao.p.mendes@inesctec.pt (J.P.M.); jmmma@utad.pt (J.M.M.M.A.); 2 Department of Physics and Astronomy, Faculty of Sciences, University of Porto, 4169-007 Porto, Portugal; 3 Chemistry Research Unit, Faculty of Sciences, University of Porto, 4169-007 Porto, Portugal; 4 Department of Physics, School of Science and Technology, University of Trás-os-Montes e Alto Douro, 5001-801 Vila Real, Portugal

**Keywords:** hydrophilic polymers, refractive index, relative humidity sensors, Fabry–Perot interferometers, long-period fiber gratings, optical fiber sensors

## Abstract

Relative humidity (RH) monitorization is of extreme importance on scientific and industrial applications, and optical fiber sensors (OFS) may provide adequate solutions. Typically, these kinds of sensors depend on the usage of humidity responsive polymers, thus creating the need for the characterization of the optical and expansion properties of these materials. Four different polymers, namely poly(vinyl alcohol), poly(ethylene glycol), Hydromed™ D4 and microbiology agar were characterized and tested using two types of optical sensors. First, optical fiber Fabry–Perot (FP) tips were made, which allow the dynamical measurement of the polymers’ response to RH variations, in particular of refractive index, film thickness, and critical deliquescence RH. Using both FP tips and Long-Period fiber gratings, the polymers were then tested as RH sensors, allowing a comparison between the different polymers and the different OFS. For the case of the FP sensors, the PEG tips displayed excellent sensitivity above 80%RH, outperforming the other polymers. In the case of LPFGs, the 10% (*wt*/*wt*) PVA one displayed excellent sensitivity in a larger working range (60 to 100%RH), showing a valid alternative to lower RH environment sensing.

## 1. Introduction

The real-time monitoring of relative humidity (RH) in scientific and industrial applications is of extreme importance, and many types of sensors were developed. Most of these sensors are based on capacitive or resistive structures that are not immune to electromagnetic radiation and are not fit to extreme and harsh environments. The usage of hydrophilic polymers in optical fiber sensors (OFS) is a thoroughly explored field of research [1,2,3,4], with most of these works using them as a functionalization layer that responds to RH variations. These polymers display a refractive index (RI) that decreases with the absorption of water molecules and exhibit considerable swelling. The changes in the polymers’ properties can be tracked by analyzing the spectral characteristics of specific optical structures such as fiber Bragg gratings (FBG) [5,6,7,8,9], Fabry–Perot interferometers (FPI) [10,11,12] or long-period fiber gratings (LPFG) [13,14], thus enabling the fabrication of optical sensors to monitor relative humidity.

Knowledge of the variation of RI and thickness of polymers with environmental parameters (such as RH) is of extreme importance for the application of optical polymers in sensors and other structures. Although several techniques were developed for the measurement of these properties in polymers [15,16], a technique for the simultaneous measurement of RI and thickness of polymer films with varying environmental parameters is here presented. Using that technique, a consistent, continuous measurement of the properties of four different polymers is reported. These RH responding polymers are poly(vinyl alcohol) (PVA), poly(ethylene glycol) (PEG), Hydromed™ D4, and microbiology Agar. These particular polymers were chosen for analysis due to the fact that they were extensively used in optical sensors, even though their properties haven’t been thoroughly studied. The work here presented provides the characterization of the optical and expansion properties of these materials, which can be of extreme importance for fabrication and optimization of relative humidity optical sensors.

Both PVA and PEG were used in relative humidity optical sensors [17,18,19,20,21,22], even though only rough measurements of their optical properties were made, which are sometimes contradictory. For the case of agar, there were several works regarding the usage of agarose (a constituent of agar) in optical applications such as sensors and probes [23,24,25], but microbiology grade agar has only had a few studies [12,26]. On the other hand, in the case of Hydromed™ D4, no measurement of the properties of this material was reported. Knowledge of the precise variation of the properties of these materials with relative humidity may allow optimization of sensor performance via simulations and adaptation of the optical structures to maximize sensitivity. Using FPI’s fabricated with these polymers, the RI and swelling properties are quantified for varying RH values. The critical deliquescence RH, which is the RH value at which a phase transition from semi-crystalline to gel state [27,28,29] was measured and was also characterized for each polymer. The polymers were then applied to LPFGs to test their performance as RH sensors, and a comparison with FPIs is made, allowing one to define which sensor and polymer combination is best for a specific context.

First, a general discussion regarding the performance of FPIs and LPFGs is presented to establish the characteristics of each optical structure. The fabrication of the polymers, the optical structures, and the method of simultaneous measurement of RI and thickness are then explained. The experimental results regarding the measurement of the polymer properties and the sensor performance are presented, followed by a discussion on both the polymer and sensor characteristics to establish which combination suits a particular context best.

## 2. Materials and Methods

### 2.1. Relative Humidity Responsive Polymers

The four different relative humidity responding polymers analyzed were PVA, PEG, a hydrogel (HydroMed™ D4), and Agar. These polymers display a considerable variation of their properties (RI and thickness) with the variation of RH, making them suitable for incorporation in RH sensors.

PVA is a water-soluble polymer that was studied extensively in the past, in particular as a humidity sensor, dehumidification agent [30,31,32] and in numerous biomedical applications [33,34] due to its ability to absorb and desorb water. Its RI was measured between 1.49 to 1.45 (at 1310 nm) [35] or between 1.49 to 1.34 (at 1550 nm) [19].

PEG is a polymer derived from petroleum containing ether linkages in its main chain, thus being referred to as a polyether. Similarly to PVA, it is a hydrophilic polymer with a large range of uses, mostly in biomedical and chemical applications due to its low toxicity and ability to absorb water [33,36]. The RI of PEG is lower than that of PVA, varying from 1.455 to 1.413 (at 960 nm) [18]. It was also used in a mixture with PVA to develop an RH sensor [22].

HydroMed™ D4 is part of a series of ether-based hydrophilic urethanes fabricated by AdvanSource Biomaterials [37]. The polymer can be dissolved in several solvents, of which ethanol was chosen. It was used previously as a sensing matrix for pH and ammonia sensors [38,39,40] and in relative humidity optical fiber sensors [17], even though its optical properties haven’t been documented.

Agar is a material derived from algae and is used in microbiology applications as a growth medium for bacteria and fungi colonies. Even though its constituents may vary from the type of agar used (depending on the colonies, different nutrients are added), it consists of a mixture of agarose and agaropectin generally in a 70–30% proportion [41,42]. Due to the different nutrients added, its RI may vary considerably. In ref. [25] the RI of bulk agarose samples was measured as a function of the agarose concentration, with values reported between 1.33 and 1.34 at 633 nm. On the other hand, in ref. [23], the RI of an agarose gel was measured with an Abbe refractometer at 1550 nm with varying RH values, displaying an RI that increases with RH from 1.455 (at 20%RH) to 1.48 (at 80%RH). Besides allowing the fabrication of RH sensors, measurement of the optical properties of agar may also provide useful information for the fabrication of optical sensors where agar may be used as a growth medium.

For the fabrication of the solutions, a solvent with low boiling point such as ethanol is preferred because it will lead to a faster evaporation and consequently the coating process of the optical structures will be faster. Nevertheless, given the low solubilities of PVA, PEG, and agar in ethanol, deionized water was used instead, which requires a longer time to fully evaporate and thus deposit the coating. In the case of hydrogel, the solvent chosen was ethanol.

Different concentrations of the polymer solutions were made to vary the thickness of the coating deposited in the LPFGs. In the case of the hydrogel, solutions with three different concentrations were produced, namely 10, 7.5 and 5% *wt*/*wt*, prepared by dissolving the high purity granules with the solvent and stirring for 2 h. The PVA solution was obtained by adding PVA to water in a concentration of 10% *wt*/*wt*, and stirring for 3 h at 60 °C. The same procedure was applied to obtain the 5 and 7.5% concentration solutions. In the case of the PEG solutions, the procedure was similar to the case of PVA, but different concentrations (50, 75 and 100% *wt*/*wt*) were used due to the low viscosity properties of PEG. For the case of agar, a 1% solution was fabricated by heating deionized water to 90 °C (agar is insoluble in water below 80 °C) and the agar was mixed by magnetically stirring for 30 min. For the fabrication of the FPIs, only the highest concentration solutions were used for each of the polymers.

### 2.2. Fabry–Perot Interferometers and Long-Period Fiber Gratings

**Figure 1:**
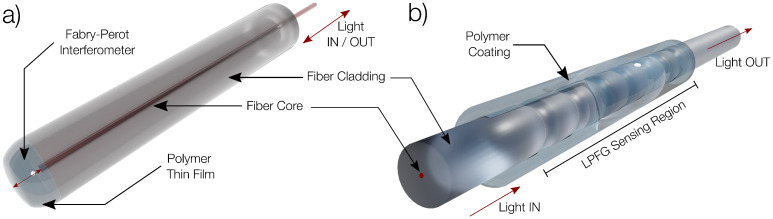
Different optical structures used for relative humidity sensing: (**a**) Inline Fabry–Perot interferometer; (**b**) Long-Period Fiber Grating.

Several solutions of different concentrations were used to produce the FPIs and the coated LPFGs, which were both fabricated in single mode fibers (Corning SMF28e). This combination of the humidity responding polymers with the fiber optical structures allows a precise monitorization of the RH of the environment. Nevertheless, due to their different characteristics, the choice of one specific structure over another can be made depending on the polymer to be used or the context in which the sensor is to be employed. Figure 1 illustrates FPIs and LPFGs OFS.

Optical fiber tip FPIs (illustrated in Figure 1a) are fabricated by dipping the tip of a cleaved fiber in the polymer and slowly removing it, thus creating a thin film after drying, which acts as the cavity. When placed in an RH varying environment, both the RI and the thickness of the polymer film will vary, creating a measurable change in the reflection spectra. These types of sensors are very compact, easy to fabricate and can be placed inside a capillary tube for protection of the polymer tip, allowing them to easily be used to monitor environmental parameters [11,12]. Another advantage of using FPIs is that the polymer used for sensing can be directly characterized, particularly the RI and thickness response to a certain environmental parameter. This process allows the optimization of the sensor and implementation of computational simulations. Thus, fabrication of optical fiber FPI tips works both as a method of characterizing the polymer and itself as a sensor.

LPFGs are illustrated on Figure 1b. They consist of a periodic modulation of the optical fiber core RI, resulting in a resonance condition that couples light from the core fiber cladding copropagating modes, creating a rejection band in the optical spectrum. Given that the coupling condition is highly dependent on the external RI, it means that the spectral features of the rejection band are highly sensitive to external RI variations. Thus, when an LPFG is coated with a humidity responding polymer, the variations in the RI of the polymer will modify the properties of the rejection band (minimum wavelength and peak attenuation), resulting in a structure that can be used as a RH sensor.

**Figure 2:**
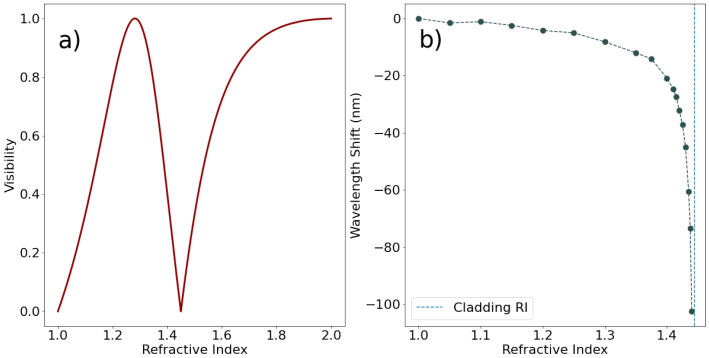
Sensitivity to variation of coating refractive index: (**a**) FPI (using an insertion loss factor *α* = 0.5); (**b**) LPFG.

LPFGs and FPIs have very different sensitivities, making them suitable for different applications. Figure 2 displays the RI response of both structures.

In Figure 2, LPFGs and FPIs display opposite behavior in the variation of the coating RI. While LPFGs display their higher sensitivity for coatings near the cladding RI, FPIs show higher variation the farther the RI is from both cores’ RI and air (because they make the two interfaces of the structure). When developing a humidity sensor, ideally the optical properties of the polymer are known, and the best optical structure can be chosen. Table 1 also shows several advantages and disadvantages of each structure.

Table 1 displays some characteristics of both FPIs and LPFGs. FPIs present several advantages such as simple fabrication and integration on a specific context, because they can be placed in a metallic capillary tube, which protects the tip and is easily placed. On the other hand, LPFGs display very high sensitivity and may allow sensing of multiple parameters, but are harder to fabricate and may require calibration of the polymers thickness, which can be difficult. After knowing the context and the characteristics of the available polymers, the optical structure chosen for the sensor can be optimized to maximize sensitivity.

### 2.3. Polymer Coating of the Optical Structures

**Figure 3:**
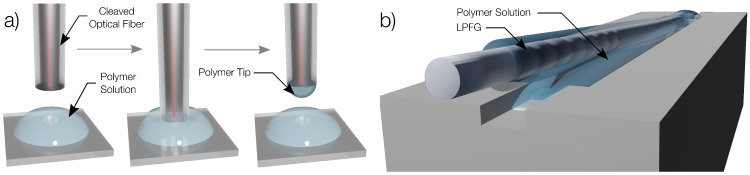
Coating process of OFS with humidity responding polymer: (**a**) FPI; (**b**) LPFG.

The FPIs were fabricated by dipping a cleaved optical fiber tip in a polymer solution, allowing the formation of a thin film when the tip is slowly pulled upwards (Figure 3a). For all polymers except agar, this process consisted of placing a drop of the solution in a microscope slide and dipping the tip of the cleaved fiber on the solution. In the case of agar, which solidifies at room temperature, the gel was heated to 80 °C to ensure that the solution is fully liquid and the microscope slide was placed on a hot plate, and the tip of the fiber was dipped in the hot solution. After fabrication, the tips were left to dry for 24 h, to guarantee the evaporation of the solvent. For each polymer, four FPIs were fabricated, to allow uncertainty measurements in the RI and thickness measurements in varying RH.

LPFGs were fabricated with the electric arc technique following the procedure described in [43,44]. The fabricated LPFGs were coated with all the polymers by stretching and dipping horizontally with a small angle between the longitudinal axes of the fiber and a U groove filled with the polymer (Figure 3b). For the case of agar, the U groove was heated at 80 °C. The fiber was then left at room temperature to ensure solvent evaporation. In this process, different concentrations of the solutions have different viscosities and thus will create thicker coatings the more concentrated the solution is, justifying why different concentrations of the various polymers were fabricated and coated different LPFGs.

### 2.4. Humidity Measurements

**Figure 4:**
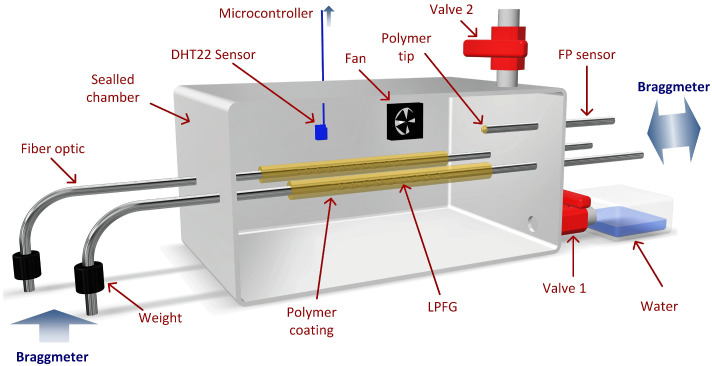
Experimental setup created to measure changes in LPFG and FPI spectra in varying values of relative humidity.

To calibrate the fabricated sensors, an experimental setup was devised in which the environment humidity could be controlled and measured (Figure 4).

The RH chamber was made from a container with two valves that connected to the exterior (where the RH was around 50%), and a side container with water connected by a valve to the main one. The purpose of these valves consists in allowing the increase and decrease of internal RH by opening the valve connected to the water container (Valve 1 in Figure 4) or the valve connected to the exterior (Valve 2 in Figure 4), respectively. A small fan was also placed inside the container to promote faster diffusion of the water molecules in the air. This setup allowed the variation of internal humidity in varying rates, depending on the fan speed.

The humidity chamber allowed for the insertion of two LPFGs at once, one with the humidity sensor and another for thermal compensation. The two fibers were placed in a stand with weights in their extremities, guaranteeing that the sensor was fully stretched. The FPIs were fixed inside the chamber, and four tips were monitored at the same time. The fibers were connected to an interrogation unit (Model FS22 Braggmeter, HBK Fibersensing, Porto, Portugal) on the outside, which recorded the spectra at all times. Also inside the container was a humidity and temperature sensor (DHT22), which has a typical accuracy of ±2%RH and ±0.5 °C and a working range of 0%RH to 100%RH and −40 °C to 80 °C [45]. This sensor was connected to a microcontroller which recorded the humidity and temperature values every 12 s.

To provide a characterization of the sensors, the LPFG spectra were taken in descending RH values. First, the valve to the side container with water was opened and the fan was turned on, ensuring that the internal RH reached around 99%RH. After this process, the valve was closed and the external one was opened, allowing a controlled decrease of RH. This procedure allowed for a slow and stable decrease of the internal RH, with each variation in 1%RH taking over two minutes. This process of slow variation of internal RH allows for the polymers to fully respond to the environmental RH changes. The spectra were recorded for every decrease in 1%RH. The data were plotted in real time to determine the spectral evolution and the working range of the fabricated sensor.

### 2.5. Simultaneous Measurement of Refractive Index and Thickness of polymers

Knowledge of the variation of the properties of the polymer is of extreme importance for the application in the optical structures, to create the optimal sensor. The advantage of using FPIs is that the RI and thickness of the polymer film can be dynamically measured with the varying external relative humidity. For each polymer, four FPI tips were fabricated, allowing to obtain uncertainty on the optical properties of the materials.

The theoretical description of these structures can be consulted in [46], and the interference spectrum is given by

(1) $$I_{R}\left(\lambda \right)=R_{1}+{\left(1-\alpha \right)}^{2}{\left(1-R_{1}\right)}^{2}R_{2}-2\sqrt{R_{1}R_{2}}\left(1-\alpha \right)\left(1-R_{1}\right)\cos\phi$$

in which α is the insertion loss factor, $R_{i}$ are the Fresnel power coefficients, defined as $R={\left(\frac{n_{1}-n_{2}}{n_{1}+n_{2}}\right)}^{2}$ (where $n_{1}$ is the RI of medium 1 and $n_{2}$ is the RI of medium 2), and $\phi =\frac{4\pi n_{pol}L}{\lambda }$, where $n_{pol}$ is the RI of the polymer, *L* is the length of the polymer cavity and λ is the wavelength.

In the case of a cavity made with a hydrophilic polymer, changes in the interference spectrum of Equation (1) will be noticeable via both the change in RI (the absorption of water decreases the RI of the cavity) and the cavity length (the cavity expands due to the water absorption). Due to the fact that Equation (1) describes a simple cosine function, it is possible to calculate the RI and cavity length of the polymer by determining the offset and amplitude of the spectrum. By comparing the experimental values of the offset and amplitude of the spectrum with Equation (1), the RI of the polymer is determined. Then, by placing the value of RI in the parameter of Equation (1) and solving for *L*, the cavity thickness is determined. This method allows for the characterization of the polymer’s response to variations in RH, thus enabling computational simulations and optimization of the sensor’s performance.

## 3. Results

### 3.1. Measurement of Refractive Index and Cavity Length

**Figure 5:**
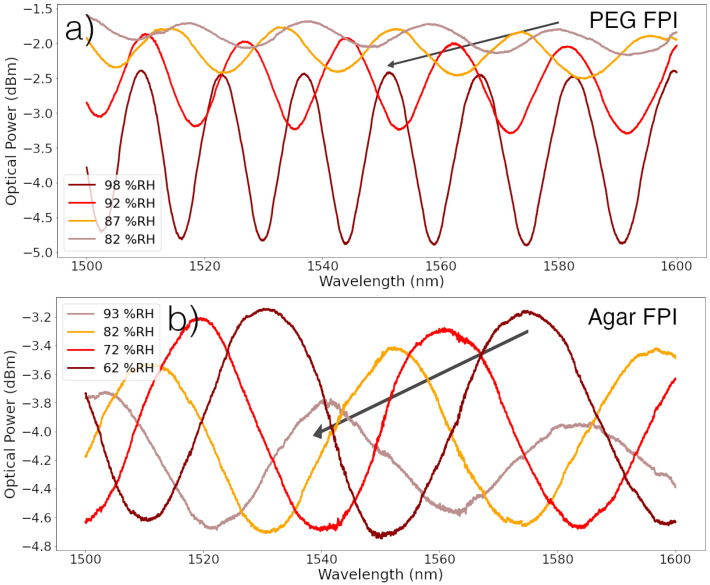
Spectra of FPI tips at different RH values, displaying considerable variation due to polymer’s response: (**a**) PEG FPI; (**b**) Agar FPI. Arrow points in direction of ascending humidity.

**Figure 6:**
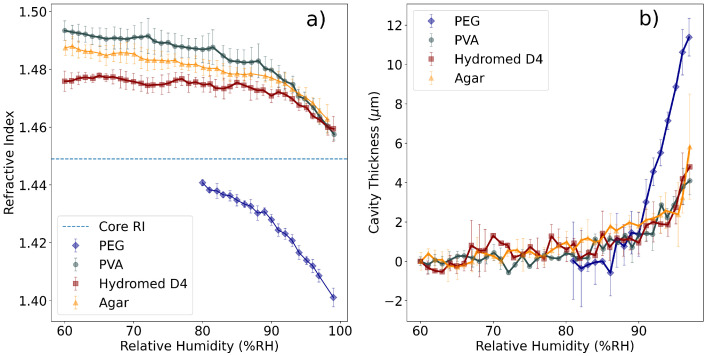
Characterization of response of various polymers to relative humidity variations: (**a**) refractive index variation; (**b**) cavity thickness variation.

Figure 5 displays the spectra registered for PEG and agar FPI tips. The response of the PEG tip differs from the agar tip. While the increase of humidity leads to a decrease of signal amplitude in the case of the agar FPI, the opposite is seen in the PEG, where the amplitude of the signal displays a clear increase with the increase of humidity. This is most likely due to the fact that PEG displays an RI below the RI of the fiber’s core, and the absorption of water will decrease the polymers RI, increasing the reflectivity of the core-polymer interface and consequently the signal’s amplitude. The opposite happens in the agar FPI, meaning that its RI is above the cores RI and water absorption decreases reflectivity. Also, it is possible to see that the signal’s period (free spectral range, FSR) increases considerably with increasing humidity for both tips, even though in the PEG tip this is much more evident. This variation is due to the absorption of water by the polymer, which expands the cavity length and decreases the FSR. It is possible to predict from Figure 5 that the PEG tips will display a much bigger swelling than the agar tips. Figure 6 shows the variation of the RI and thickness of the polymers, measured using the procedure described in Section 2.5.

Figure 6 displays the variation of both RI and cavity thickness of the three polymers tested. (a) shows the three polymers displaying different RI values, making them suitable for different uses. The observations made in Figure 5 are clear, namely the fact that PEG displays an RI below the core’s RI (as opposed to agar) and that it displays swelling effects considerably larger than the other polymers.

All polymers display a phase transition from a solid semicrystalline state to a gel state at a specific RH value, which is called the deliquescence relative humidity (DRH). In the case of PVA, agar, and the hydrogel, this transition is seen at approximately 90%RH, where it is possible to see an abrupt decrease of RI and increase of film thickness. On the other hand, in the case of PEG this transition is seen at approximately 80%RH, which is in agreement with [18]. In this case, the swelling caused by the absorption of water is larger than in the cases of PVA and hydrogel, leading to a very large deformation of the polymer cavity.

**Figure 7:**
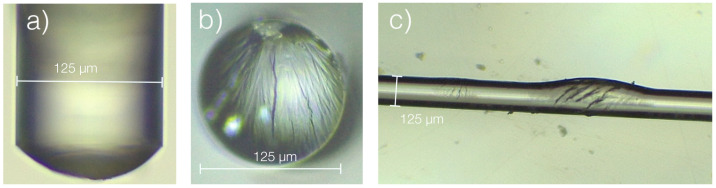
Degradation of multiple PEG films caused by shrinking at low RH: (**a**) FPI tip, side view; (**b**) FPI tip, front view; (**c**) optical fiber coated with PEG film.

Below the DRH, it was not possible to retrieve any information from the spectra of the PEG FPIs. When analyzing the tips on a microscope, it was possible to verify that there were significant defects (Figure 7), explaining why the RI and cavity thickness values below 80%RH could not be determined.

Figure 7a,b shows the defects seen in the PEG FPI tips after the RH run. Also, in Figure 7c a simple optical fiber was coated with a high concentration PEG solution, to create a thick PEG layer to test the effect of RH variations. Structures fabricated with PEG coatings of several micrometers (such as FPIs) are unsuitable for sensing applications below the DRH, due to the appearance of defects in the film caused by the abrupt phase transition seen in Figure 6. On the other hand, several cycles were performed for the hydrogel, PVA, and agar tips with no clear degradation seen, meaning that these polymers could be used on both FPIs and LPFGs.

Table 2 summarizes the results of the characterization of the different FPI tips. Several conclusions can be drawn from the polymers properties, starting with the fact that PEG shows very good results to be used in contexts where the RH is always higher than 80%RH, because both RI and thickness variations show very high sensitivities, making it an ideal polymer for high RH monitoring. On the other hand, regarding the properties of the other three polymers, PVA and agar display a higher RI variation in the region below DRH, while the hydrogel flattens out below 90%RH (Figure 6). This leads to the conclusion that for RH monitoring in lower humidity contexts, PVA and agar sensors could display more advantages than the hydrogel. On the other hand, the stability of the optical properties of the hydrogel could be a desirable feature when it is used as a sensing matrix. Regarding the expansion properties, agar displays a slightly bigger expansion rate in the region below DRH, while PVA and the hydrogel have similar values.

In principle, all polymers could be used as coatings on LPFGs, but in different regimes. In the case of PEG, the RI values seem to lie below the RI of the cladding of an optical fiber, meaning that (at least in the 80% to 100%RH regime) this polymer will not induce transitions from guided to leaky modes of the cladding, and that a thick coating could be employed. In the case of PVA, agar and hydrogel, the RI is clearly above the cladding RI, meaning that only very thin overlays could be applied to an LPFG. This process makes the fabrication of LPFG sensors with these polymers considerably harder, because control of the thickness of the deposited layer is very difficult.

### 3.2. Fabry–Perot Interferometers as Relative Humidity Sensors

Regarding the performance of the FPIs as RH sensors, the visibility was chosen as the figure of merit. In an FPI, the visibility is defined as (with $P_{max}$ and $P_{min}$ and the maximum and minimum values of optical power, respectively):

(2) $$V=\frac{P_{max}-P_{min}}{P_{max}+P_{min}}$$

**Figure 8:**
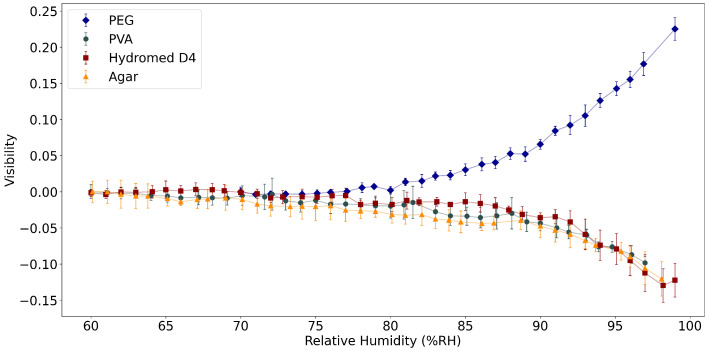
Variation of visibility with relative humidity of various FP tips for PVA, PEG, and hydrogel, normalized to visibility at 60%RH.

Comparing Equation (2) with Equation (1), it is possible to see that the visibility is dependent only on $n_{pol}$ and α. Figure 8 shows the visibility variation of the three polymers from 60% to 100%RH.

Figure 8 shows the different response of the visibility of the FP tips, normalized at 60%RH. The variation is as expected from Figure 6a. The PEG tip increases its visibility with larger values of RH because $n_{PEG}<n_{core}$, and the increase in RH further distances the two RI values, making PEG a good choice for RH sensing at high RH values (above 80%RH). In the case of PVA, agar and the hydrogel, the opposite happens: the increase of RH leads to the RI of both polymers being closer to $n_{core}$, thus justifying the decrease in visibility. Again, the transition from solid to gel state is clearly seen, and in the gel state the sensitivity to RH variations is increased. Below DRH (approximately 90%RH), the sensitivity of the polymers applied to the FPIs is much lower.

### 3.3. Long-Period Fiber Gratings as Relative Humidity Sensors

The results shown in Figure 6 display the existence of two clear groups of polymers (grouped by similar properties). The first of these groups is composed of PVA, agar and hydrogel, which display similar RI and thickness variations under changes in RH; the second is composed of PEG, which displays very high variations under RH changes above DRH. These polymers were then applied to LPFGs to test them as RH sensors.

**Figure 9:**
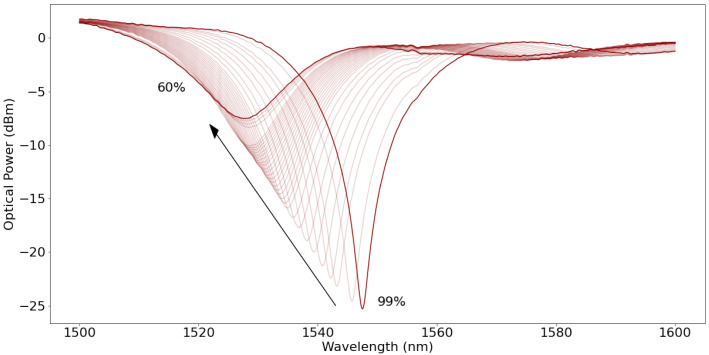
Variation of 10% (*wt*/*wt*) PVA-coated LPFG spectra with varying RH. Arrow points in direction of descending humidity.

Notice the sensitivity to external RH variations seen in Figure 2b, which shows that near an external medium with $n_{pol}\approx n_{clad}$ the sensitivity will be high. This provides the ideal context for the application of PEG above DRH, because its RI is always below but close to $n_{clad}$. On the other hand, the other polymers have RI above $n_{clad}$, meaning that the thickness of the deposited layer will have to be controlled. If the evanescent field of the cladding modes is fully contained within the polymer layer, the modes will not be guided and will become radiative, displaying poor sensitivity to external RI changes. On the other hand, if the thickness of the deposited layer is controlled, the sensor fabricated with either PVA and agar could work with high sensitivity below DRH, because the RI response of these polymers shows some variation from 60 to 90%RH, as opposed to the hydrogel, which is approximately flat in this region, see Figure 6. Figure 9 displays the collected spectra for the 10% (*wt*/*wt*) PVA coated LPFG at varying values of RH.

**Figure 10:**
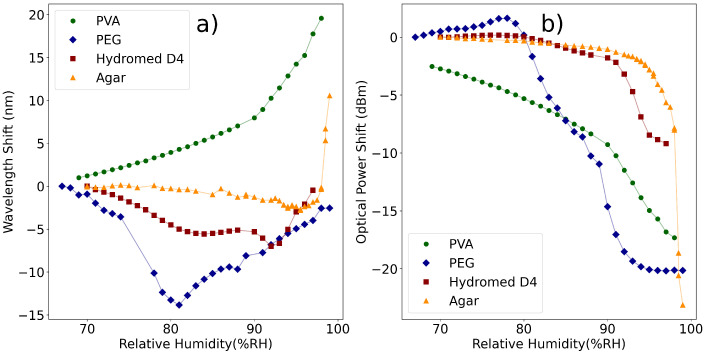
Response of different polymer-coated LPFG sensors: (**a**) Wavelength Shift; (**b**) Optical Power Shift.

In Figure 9, the good performance seen in the spectra was only possible to achieve by coating the LPFG with several polymer thicknesses (by varying the concentration of the solution) and testing the LPFG. As stated above, for each polymer several solutions of different concentrations were made and deposited on LPFGs. Given that all other fabrication parameters are kept as constant as possible, the variation of the coating thickness on the LPFG can be attributed to the variation of solution concentration only. This process allows the optimization of the sensor’s performance by controlling the thickness of the layer deposited, which is important in polymers with RI above the cladding RI. Figure 10 shows the comparison of the best performing LPFG sensor for each polymer and Table 3 summarizes the results seen in Figure 10.

Of the four sensors presented, the PVA (at 10% *wt*/*wt*) displays the best performance, showing no dead zones, a working range from 60%RH to 100%RH and two regions of different sensitivity, below 90%RH (the DRH of the polymer) and above that value. Above DRH, the sensitivity is further increased (from 0.290±0.006 to 1.03±0.08 nm/%RH in wavelength shift and from −0.275±0.007 to −0.84±0.04 dB/%RH in optical power shift), which is expected from the polymers characteristics seen in Figure 6. The sensor displays high sensitivity in both wavelength shift and optical power shift, making it an ideal sensor.

The PEG sensor fabricated with a 75% (*wt*/*wt*) solution also displays interesting properties. Above DRH (80%RH), the wavelength shift response shows a linear region to approximately 100%RH, with high sensitivity (0.58±0.02 nm/%RH). In optical power shift, a good sensitivity is also attained (−1.48±0.08 dB/%RH), but displaying dead zones below 80%RH and above 95%RH.

For the case of the hydrogel and agar LPFGs, the performance obtained was below the other cases. The hydrogel LPFG, coated with a 7.5% (*wt*/*wt*) solution, shows a good response in optical power shift from 80%RH to 100%RH (−0.23±0.04 dB/%RH), but falls short under comparison with the PEG LPFG. In the case of the agar LPFG, no optimization of the sensor’s concentration was possible. This is due to increased free variables in the fabrication which affect coating thickness, because the agar needs heating to 80 °C to become a gel and allow the coating of the LPFGs. Variations in the temperature of the polymer may influence viscosity, which in turn makes thickness control harder. Given the fact that PVA displays similar properties to these polymers (as can be seen in Figure 6), it is recommended over the hydrogel and agar for LPFG coating, due to easier fabrication and good RI variation under DRH.

## 4. Discussion

The determination of the optical and expansion properties of the different polymers used do not always agree with works published previously. In previous publications, the RI of PVA was measured using a calibrated side polished fiber coating [35] at 1310 nm, reporting a linear variation of RI from approximately 1.49 (below 30%RH) to 1.45 (at 100%RH); or a tilted fiber Bragg grating [19], reporting a variation from 1.49 (below 20%RH) to 1.34 (at 98%RH) at approximately 1550 nm. The results here presented are in agreement with [35], assuming that there is negligible chromatic dispersion from 1310 nm to 1550 nm, which is not unusual in many materials.

The PEG measurements were also in agreement with previous publications [18], in which the RI of PEG films was measured at 632.8 nm using a prism configuration. In this work, the RI of PEG was reported to vary from 1.452 (at 13%RH) to 1.413 (at high RH values). These results are in qualitative agreement (due to chromatic dispersion) with the results presented in Figure 6. In ref. [18], the reported DRH was also around 80%RH, the same as reported in this work.

Regarding the measurement of the optical properties of agar, they show considerable difference from values reported for the agarose gels. In ref. [25], the low RI reported could be due to the absence of agaropectin, which is the other constituent of agar. This result could also prove useful for optical RH sensors, depending on whether a low RI (agarose) or high RI (agar) polymer is needed. On the other hand, the measurements reported in [23], which show that the RI of agarose increases from 1.45 to 1.48 with the increase of external RH, are in disagreement with the measurements here reported. In general, RH-sensitive polymers and gels decrease their RI with the absorption of water, as shown in Figure 6.

In the case of the hydrogel, no previous works reporting the optical properties of this material were found.

The results described previously allow for the comparison between the different sensors and the different polymers analyzed. Regarding the FPIs, PVA, agar and hydrogel FPIs display a very similar response, while the PEG displays an increase in visibility with increase of external RH (which is explained by Figure 6, because the RI of PEG is getting further away from the RI of the core of the fiber). In this case, the PEG tip displays a higher sensitivity than the other polymers and a working range between 80%RH and 100%RH (above DRH). In the other polymers, the FPIs visibility shows almost no variation below 80%RH, making PEG the most suitable choice for high-sensitivity, high-RH measurements.

Regarding the LPFGs, the results obtained in the 10% *wt*/*wt*. PVA coated LPFGs show that this sensor has the best performance by far in both wavelength shift and optical power shift, in the range of 60%RH to 100%RH, with no drawbacks seen. The RI curve measured for PVA (Figure 6) indicates that the RI is considerably higher than the optical fiber cladding RI, meaning that the coating deposited on the fiber is most likely of the order of hundreds of nanometers and repeatability could be hard to obtain. The results presented demonstrate that it is possible to fabricate a high-sensitivity RH sensor with an LPFG, but future work is needed to develop sensors with coatings of controlled thickness, which is essential in these types of sensors.

## 5. Conclusions

The RI and thickness response of PVA, PEG, agar, and Hydromed D4 were analyzed and quantified using a dynamical method of measurement. This process allowed for the characterization of the RH response of each polymer and a discussion of which polymer may be used in two different optical sensors, Fabry–Perot Interferometers and Long-Period Fiber Gratings. For the case of FPIs, the results showed that PEG is a suitable choice for high RH sensing due to its response above deliquescence RH. In the case of LPFGs, the PVA one displays suitable properties for RH sensing, mostly due to its variations of optical properties below DRH. In the case of Hydromed D4, the optical stability was demonstrated, making it a suitable choice for sensing matrix in other applications.

## Figures and Tables

**Table 1:** Comparison of properties of FPIs and LPFGs.

	Fabry–Perot Interferometers	Long-Period Fiber Gratings
Fabrication	Very simple	Harder
Equipment	Reflection Mode interrogation	Transmission Mode interrogation
Integration	Easy in capillary tube	Harder in solid environments
Sensitivity	Low for polymers near $n_{core}$	High (adjusting film thickness)

**Table 2:** Characteristics of different polymers with variation of relative humidity.

Polymer	Refractive Index Sensitivity (RIU/%RH)	Expansion Coefficient (μm/%RH)	Deliquescence Relative Humidity	Observations
PVA	$\left(-4.0\pm 0.2\right)\times 10^{-4}$	0.033±0.007	90%RH	Below DRH
PEG	$\left(-2.9\pm 0.1\right)\times 10^{-3}$	1.58±0.05	80%RH	Above DRH
Hydrogel	$\left(-1.4\pm 0.2\right)\times 10^{-4}$	0.040±0.008	90%RH	Below DRH
Agar	$\left(-3.6\pm 0.1\right)\times 10^{-4}$	0.064±0.006	90%RH	Below DRH

**Table 3:** Sensitivities of different polymer-coated LPFGs in both Wavelength and Optical Power.

Polymer	Working Range (%RH)	Wavelength Sensitivity (nm/%RH)	Optical Power Sensitivity (dB/%RH)
PVA	65–90 90–100	0.290±0.006 1.03±0.08	−0.276±0.007 −0.84±0.04
PEG	65–80 80–100	−0.8±0.2 0.58±0.02	- −1.43±0.08
Hydrogel	65–95	−0.22±0.03	−0.23±0.04
Agar	65–95	−0.100±0.005	−0.089±0.005

## Data Availability

Not applicable.
